# Mode of inheritance for biochemical traits in genetically engineered cotton under water stress

**DOI:** 10.1093/aobpla/plw008

**Published:** 2016-02-02

**Authors:** Muhammad Ali Abid, Waqas Malik, Azra Yasmeen, Abdul Qayyum, Rui Zhang, Chengzhen Liang, Sandui Guo, Javaria Ashraf

**Affiliations:** 1 Genomics Lab, Department of Plant Breeding and Genetics, Faculty of Agricultural Sciences and Technology, Bahauddin Zakariya University, Multan 60000, Pakistan; 2 Biotechnology Research Institute, Chinese Academy of Agricultural Sciences, 100081 Beijing, China; 3 Department of Agronomy, Faculty of Agricultural Sciences and Technology, Bahauddin Zakariya University, Multan 60000, Pakistan

**Keywords:** Biochemical markers, carotenoids, Cry1Ac toxin, enzymatic antioxidants, non-additive gene action

## Abstract

Drought is an abiotic environmental stress that can significantly reduce crop productivity. We examined the mode of inheritance for different biochemical traits including total soluble proteins, chlorophyll *a*, chlorophyll *b*, total chlorophyll, carotenoids, total phenolic contents and enzymatic antioxidants (superoxide dismutase, peroxidase and catalase), and their relationship with *Bacillus thuringiensis* (*Bt*) toxin under control and drought conditions. Eight genetically diverse cotton genotypes were selfed for two generations to ensure homozygosity. Fifteen F_1_ hybrids were developed by crossing five non-*Bt* female lines with three *Bt* male testers. The F_1_ hybrids and eight parents were finally evaluated under control (100 % field capacity (FC)) and drought (50 % FC) conditions in 2013. The biochemical traits appeared to be controlled by non-additive gene action with low narrow sense heritability estimates. The estimates of general combining ability and specific combining ability for all biochemical traits were significant under control and drought conditions. The genotype-by-trait biplot analysis showed the better performance of *Bt* cotton hybrids when compared with their parental genotypes for various biochemical traits under control and drought conditions. The biplot and path coefficient analyses revealed the prevalence of different relationships between Cry1Ac toxin and biochemical traits in the control and drought conditions. In conclusion, biochemical traits could serve as potential biochemical markers for breeding *Bt* cotton genotypes without compromising the optimal level of *Bt* toxin.

## Abstract (precis)

The optimal level of *Bt* toxin in *Bt* cotton is imperative for sustainability and adoption of *Bt* cotton under water stressed and non-stressed environments. We investigated the mode of inheritance and association of various drought tolerance biochemicals traits with *Bt* toxin under normal and water stressed conditions. We observed non-additive gene action coupled with low heritability estimates for all studied biochemical traits. The different kinds of association between *Bt* toxin and biochemical traits proved to be a simple innovative strategy. Furthermore, it is concluded that different biochemical traits can serve as a potential biochemical markers in future for breeding drought tolerant *Bt* cotton.

## Introduction

Plants are more vulnerable to unfavourable environmental conditions during growth, development and reproduction due to their sessile nature (Trewavas 2002). Drought is one of the major factors limiting crop production and commonly leads to substantial losses in yield. Plants have evolved a variety of different mechanisms at morphological, physiological, cellular and biochemical levels to overcome water stress conditions (Fang and Xiong 2015). In addition to naturally occurring mechanisms, more than 80 years of breeding activities have led to an increase in crop yield under drought conditions. Although fundamental research has provided considerable gains in our understanding of the responses of plants to water deficits, there is still a large gap between yields of crops in stress and non-stress environments. Minimizing the ‘yield gap’ and increasing yield stability under different water-deficient conditions are of strategic importance for plant scientists (Cattivelli *et al*. 2008).

Water stress leads to the production of reactive oxygen species (ROS), and their accumulation causes toxicity, peroxidation of cellular membranes, oxidation of carbohydrates, proteins, lipids and even DNA (Apel and Hirt 2004; Shah *et al.* 2011). The balance between ROS generation and scavenging is maintained by various enzymatic antioxidants like superoxide dismutase (SOD), catalase (CAT), peroxidases (PODs) and non-enzymatic antioxidants including phenolics, ascorbate, carotenoids and tocopherols (Gill and Tuteja 2010). Superoxide dismutase, POD and CAT comprise the main enzymatic antioxidant system that catabolize free radicals and limit the potential for oxidative damage (Apel and Hirt 2004). Superoxide dismutase catalyses the superoxide (O_2_^−^) to O_2_ and H_2_O_2_, then POD and CAT catalyse the conversion of H_2_O_2_ to H_2_O and O_2_ (Mittler *et al.* 2004). In addition to this, leaf water potential influences the photosynthetic process by reduction in CO_2_ fixation due to stomatal closure (Flexas *et al.* 2004), disturbing photosynthetic pigments like chlorophyll and carotenoids and damaging the photosynthetic apparatus (Wahid and Rasul 2005; Parida *et al.* 2007). The concentration of *Bacillus thuringiensis*(*Bt*) protein in different transgenic crops including cotton is significantly influenced by water deficit conditions (Wang *et al.* 2001; Luo *et al.* 2008). Reduction in efficacy of *Bt* crystalline endotoxins in genetically engineered crop due to abiotic stresses would result in poor control over targeted pests and may increase resistance against *Bt* proteins (Tabashnik *et al.* 2008).

The interaction of *Bt* toxin production and water stress could be particularly important to determine whether transgenic crops will continue to be effective against target insects/pests in the future. Therefore, information regarding the inheritance of stress-related traits could be helpful for plant breeders to devise a breeding strategy. The line × tester analysis provides better estimates for genetics components, detection of suitable parents and superior crosses needed for selection procedures in further generations (Ganapathy *et al.* 2005; Ahuja and Dhayal 2007). Selection for the improvement of specific trait in an earlier segregating population would be effective if the trait of interest is controlled by additive effects. However, in cases of greater proportion of non-additive (epistatic and dominant) gene effects, the selection should be carried out in later generations (Jagtap 1986). Genes with additive effects would result in increased general combining ability (gca), and non-additive gene action is responsible for specific combining ability (sca). Further, the selection procedure could be more effective by investigating the nature of association among traits, often leading to decisive results about breeding of plants for a specific purpose (Cakmakci *et al.* 1998). In this regard, biplot and path coefficient analyses are reliable biometrical techniques (Dewey and Lu 1959; Yan and Kang 2003).

Transgenic cotton expressing *Bt* toxin has been the most rapidly adopted genetically engineered crop worldwide (James 2002; Barwale *et al*. 2004; Dong *et al.* 2005). This insect-resistant cotton is effective in controlling lepidopteran insects and benefits farmers and the environment by reducing the synthetic insecticidal sprays and preserving the population of beneficial arthropods (Gianessi and Carpenter 1999; Tabashnik *et al*. 2002). The sustainability of this technology depends largely upon adequate concentration of *Bt* protein during the entire growth period of plants. However, expression of the *Bt* transgene is affected by water stress environment (Traore *et al*. 2000). Keeping in mind the increasing shortage of water in the world and its impact on cotton production, it is imperative to investigate the genetic pattern of various biochemical traits for drought tolerance in cotton. The objective of this study was to investigate the mode of inheritance and nature of association among various biochemical traits in interspecific and intraspecific crosses of cotton under normal and drought conditions. These findings would pave the way for cotton breeders to develop drought-tolerant *Bt* cotton varieties.

## Methods

### Plant materials

The plant materials comprised eight genetically diverse cotton genotypes. Five genotypes belonging to *Gossypium hirsutum* (SA-1357, MNH-814, VH-303, MNH-886 and FH-142) and one belonging to *G. barbadense* (GIZA-7) have white fibre colour and were collected from the Cotton Research Station in Multan, Pakistan. Two genotypes belonging to *G. hirsutum* (BZUG1 and BZUB) have green and brown fibre colour, respectively, and were collected from the Department of Plant Breeding and Genetics, Bahauddin Zakariya University, Multan, Pakistan.

### Development of breeding materials

The genotypes were selfed for two generations during 2011–12 to ensure their purity. Four seeds of each selfed genotype were sown in earthen pots (40 cm diameter and 75 cm deep) containing 16 kg of sandy loam soil during November 2012, in a glasshouse having automatic temperature controls. Temperatures of 30 °C ± 5 and 20 °C ± 5 were maintained during day and night, respectively. At the 15th day of emergence, plants were thinned, maintaining only two healthy seedlings per pot. The recommended cultural practices were adopted during the conduct of experiment. Lines × Testers (5 × 3) crosses were made at the flowering stage. All the non-*Bt* genotypes, i.e. SA-1357, MNH-814, BZUG1, BZUB and GIZA-7, were used as female parent (lines), while three *Bt* genotypes MNH-886, FH-142 and VH-303 served as male parent (testers). Crossed bolls were hand-picked and ginned using a single-ruler ginning machine to derive F_0_ seed.

### Evaluation of breeding materials

In May 2013, four F_0_ seeds from each of the 15 crosses along with their parents were sown in plastic pipes (90 cm depth and 3 cm diameter) in two sets (i.e. control and drought) in a glasshouse. Temperatures of 30 °C ± 5 day and 20 °C ± 5 night were maintained using the automatic cooling and heating systems of the glasshouse. Clay loam and farmyard manure in a ratio of 3 : 1 were used as media in pipes to facilitate plant growth. The experiment was laid out in a completely randomized design with three replications and each replication comprised five pipes. The same amount of water was given to the both sets of plants, and thinning was carried out at the 15th day of emergence to have only one plant per pipe. After the 15th day of plant emergence, two different levels, 100 % field capacity (control) and 50 % field capacity (drought), were maintained on a gravimetric basis (Nachabe 1998). These field capacity levels were maintained up to harvesting.

### Sample collection

The fully expanded leaf samples from plants of both control and drought treatments were collected at 90 days after emergence because at this stage, the plant has a maximum number of developing bolls and an optimum amount of *Bt* toxin is very necessary, along with other biochemical traits, to avoid boll worm attack and drought stress. The collected leaf samples were stored immediately at –80 °C for different biochemical analyses. All the spectrophotometric analyses of biochemical traits were conducted using Implen-Nanophotometer (Germany) in the Genomics Lab at the Department of Plant Breeding and Genetics, Bahauddin Zakariya University, Multan, Pakistan.

### Determination of total soluble proteins

For the extraction of total soluble proteins (TSP, mg g^−1^), 0.5 g of leaf sample was ground in 1 mL of 50 mM phosphate buffer with pH 7.2. The ground material was centrifuged at 12 000 r.p.m. for 5 min and supernatant was transferred to another 1.5 mL centrifuged tube. Bradford assay (Bradford 1976) was used to quantify the TSP by constructing a standard curve (10, 20, 30, 40 and 50 µg mL^−1^) for reaction mixture of bovine serum albumin, dye stock (Coomassie Brilliant Blue G-250 dye) and distilled water. The absorbance of reaction mixture for the standard curve and that of the sample was recorded at 595 nm.

### Determination of leaf chlorophyll and carotenoid contents

Leaf carotenoids, chlorophyll (*a* and *b*) and total chlorophyll contents were analysed by grinding 0.5 g of the leaf sample in 80 % acetone solution followed by filtration through Whatman #1 paper. The absorbance of filtrate was recorded at 663, 644 and 452.5 nm. The contents of chlorophyll *a*, chlorophyll *b*, total chlorophyll and carotenoids were calculated (µg mL^−1^) according to formulae given by Metzner et al. (1965).

Chlorophylla=(10.3×E663)−(0.98×E644)

Chlorophyllb=(19.7×E644)−(3.87×E663)

Total chlorophyll=chlorophylla+chlorophyllb

Carotenoids=4.2×E452.5−{(0.0264×chlorophylla)+(0.426×chlorophyllb)}

where *E* is the absorbance at that specific wavelength.

### Total phenolic contents

The total phenolic contents (TPC, mg GAE g^−1^) of leaf samples were quantified according to Ainsworth and Gillespie (2007). Gallic acid solutions of different concentrations (500, 250, 150 and 100 mg L^−1^) were prepared to plot the calibration curve by determining absorbance at 760 nm. For preparation of the sample, 0.5 g of cotton leaf was ground in 80 % acetone solution followed by filtration through Whatman #1 paper. The volume of filtrate was increased to 10 mL by adding acetone solution. For preparation of the reaction mixture, a 20 μL sample or standard was added in 100 μL of Follin–Ciocalteu reagent, 1.58 mL of distilled water within 8 min and mixed with 300 μL of 20 % (w/v) sodium carbonate solution. The prepared reaction mixture was kept in darkness for 2 h. Total phenolic contents of samples were determined at 760 nm.

### Determination of enzymatic antioxidants

For preparation of the enzyme extract, a 0.5 g leaf sample was ground in 5 mL of 50 mM phosphate buffer, pH 7.8. The extract was centrifuged at 15 000 r.p.m. for 20 min and supernatant was transferred to separate 1.5 mL tube and kept in darkness.

#### Superoxide dismutase EC number (1.15.1.1)

Superoxide dismutase activity was determined following Giannopolitis and Ries (1977) using its ability to inhibit the photochemical reduction of nitrobluetetrazolium (NBT) at 560 nm. The reaction mixture was primed by mixing 50 μL of enzyme extract, 1 mL of 50 μM NBT, 500 μL of 75 mM ethylenediaminetetraacetic acid, 950 μL of 50 mM phosphate buffer, 1 mL of 1.3 μM riboflavin and 500 μL of 13 mM methionine. Test tubes containing the reaction mixture were incubated under 30 W fluorescent lamp illuminations for 5 min. The reaction was stopped when the fluorescent lamp was switched off and covered with aluminium foil. Test tubes containing the same reaction mixture without enzyme extract served as blank. Blue formazan was developed due to photoreduction of NBT, which was measured using absorbance at 560 nm. Superoxide dismutase activity was expressed as IU min^−1^ mg^−1^ of protein.

#### Peroxidase EC number (1.11.1.7)

Peroxidase activity (mmol min^−1^ mg^−1^ protein) was determined according to the method described by Chance and Maehly (1955). Peroxidase activity was determined by guaiacol oxidation, and the unit of POD was defined as 0.01 absorbance change min^−1^ mg^−1^ protein. The 3 mL reaction mixture was prepared by mixing 100 μL of enzyme extract, 2 mL of 50 mM phosphate buffer, 500 μL of 40 mM H_2_O_2_ and 400 μL of 20 mM guaiacol. The change in absorbance was recorded at 470 nm for every 20 s up to 5 min.

#### Catalase EC number (1.11.1.6)

Catalase activity was estimated according to Chance and Maehly (1955), which involved the initial decomposition of H_2_O_2_. The 3 mL reaction mixture for the determination of CAT contained 2 mL of 50 mM phosphate buffer, 900 μL of 5.9 mM H_2_O_2_ and 100 μL of enzyme extract. Absorbance was observed for every 30 s to 5 min at 240 nm. The unit of CAT activity was defined as decomposition of μmol of H_2_O_2_ min^−1^ mg^−1^ protein.

### Cry1Ac protein concentration assay

The concentration of Cry 1Ac (in µg g^−1^) in cotton leaf extracts was determined through enzyme-linked immunosorbent assay following Shan *et al.* (2007). An ice-cold 1× sample extraction buffer (500 μL) was used to homogenize the lyophilized tissue. The lyophilized tissue was macerated through mortar-driven pestle at 3000 r.p.m. for 30 s, then chilled on ice for 30 s and macerated for 30 s again, centrifuged at 8000 r.p.m. for 15 min. Then the supernatant was collected for the determination of Cry1Ac protein. The antibody, buffer blank, standards and controls (negative and positive) were added to each well and incubated at 37 °C. After 45 min, the buffered enzyme was added and incubated for 30 min at room temperature. Finally, the absorbance was recorded at 405 nm.

### Statistical analysis

The data for all biochemical traits, i.e. TSP, chlorophyll *a*, chlorophyll *b*, carotenoids, total chlorophyll, TPC and enzymatic antioxidants (SOD, POD and CAT) under both control and drought conditions, were analysed following the line × tester analysis (Singh and Chaudhary 1999). The sum of square for genotypes was subdivided into variation among parents, among parents vs. crosses and among crosses. The sum of square for parents was also subdivided into variation among lines, among testers and among line × testers.

#### Estimation of variance components and heritabilities

The estimates of variance for combining abilities, genetic components and heritabilities were calculated using the mean square values. The variances due to gca and sca were tested against their respective error variances, derived from the analysis of variance of the different traits as follows:

$$\text{I}.\;\text{Covariance of half sib line}=\text{Cov}.\text{H}.\text{S}.\;(\text{line})=\frac{\text{M}{\text{s}}_{l}-\text{M}{\text{s}}_{l\times t}}{rt}$$

$$\;\begin{array}{ll} \text{II}.\; & \text{Covariance of half sib tester}=\text{Cov}.\text{H}.\text{S}.\;(\text{tester})\\ & \;=\frac{\text{M}{\text{s}}_{t}-\text{M}{\text{s}}_{l\times t}}{rl} \end{array}$$

$$\;\begin{array}{ll} \text{III}.\; & \text{Covariance of half sib}\;(\text{average})\\ & \;=\frac{1}{r(2lt-l-t)}\left\{\frac{\left(l-1)(\text{M}{\text{s}}_{l})+(t-1)(\text{M}{\text{s}}_{t}\right)}{l+t-2}-\text{M}{\text{s}}_{l\times t}\right\} \end{array}$$

$$\;\begin{array}{ll} \text{IV}\text{.} & \;\;\text{Covariance of full sib}\\ & \;\;\;=\frac{\left(\text{M}{\text{s}}_{l}-\text{M}{\text{s}}_{e})+(\text{M}{\text{s}}_{t}-\text{M}{\text{s}}_{e})+(\text{M}{\text{s}}_{l\times t}-\text{M}{\text{s}}_{e}\right)}{3r}\\ & \;+\frac{6r\text{Cov}.H.S.-r(l+t)\text{Cov}.H.S.}{3r} \end{array}$$

where *l*, *t*, *r*, MS_l_, MS_t_, MS_l×t_ and MS_e_ are number of lines, number of testers, number of replications, mean square of lines, mean square of testers, mean square of line × tester and error mean square, respectively.

General combining ability variance and sca variance were calculated following the formulae

$$\text{V}.\;\sigma_{\mathrm{gca}}^{2}=\text{Cov}.\text{H}.\text{S}.=\left(\frac{1+F}{4}\right)\sigma_{A}^{2}=\frac{1}{2\sigma_{A}^{2}}$$

So, $\sigma_{A}^{2}=2\sigma_{\mathrm{gca}}^{2}$

$$\;\text{VI}.\;\sigma_{\mathrm{sca}}^{2}={\left(\frac{1+F}{2}\right)}^{2}\sigma_{D}^{2}=\sigma_{D}^{2}$$

So, $\sigma_{D}^{2}=\sigma_{\mathrm{sca}}^{2}$

Additive and dominance genetic variances were calculated by taking inbreeding coefficient as one (*F* = 1). Narrow sense heritability (*h*^2^) was calculated using the formula

$$\text{VII}.\;h^{2}=\frac{\sigma_{A}^{2}}{\sigma_{P}^{2}}$$

#### Per cent contribution of lines, testers and lines × testers

$$\begin{array}{ll} \text{I}.\; & \text{Per cent contribution of lines}\\ & \;=\frac{\text{Sum square of lines}}{\text{Sum square of crosses}}\times 100\\ \text{II}.\; & \text{Per cent contribution of tester}\\ & \;=\frac{\text{Sum square of testers}}{\text{Sum square of crosses}}\times 100\\ \text{III}.\; & \text{Per cent contribution of lines}\;\times \;\text{testers}\\ & \;=\frac{\text{Sum square of lines}\;\times \;\text{testers}}{\text{Sum square of crosses}}\times 100 \end{array}$$

#### Estimation of combining ability effects

General combining ability and sca were calculated from the two-way table of lines vs. testers in which each value was total over replications (Singh and Chaudhary 1999)

$$\;\text{I}.\;\text{gca effects of}i\text{th}\;\text{line}=gi=\frac{xi..}{tr}-\frac{x\ldots }{ltr}$$

$$\;\text{II}.\;\text{gca effects of }j\text{th}\;\text{tester}=gj=\frac{x.j.}{lr}-\frac{x...}{ltr}$$

$$\text{III}.\;\text{sca effects of}i\text{th}\;\text{cross}=sij=\frac{xij}{r}-\frac{xi..}{tr}-\frac{x.j.}{lr}-\frac{x\ldots }{ltr}$$

where *l* is the number of lines, *t* the number of testers, *r* the number of replications, *xi*.. the sum of *i*th line over all testers and replications, *x*… the sum of means of all crosses of lines and testers over replications, *x.j.* the sum of *j*th tester over lines and replications, *xij* the sum of mean *ij*th hybrid combination over replications.

#### Estimation of standard error for combining ability effects

$$\;\text{I}.\;\text{SE}(gi)\;\text{lines}={\left(\frac{\text{MSe}}{tr}\right)}^{1/2}$$

$$\;\text{II}.\;\text{SE}(gi-gj)\;\text{lines}={\left(\frac{2\text{MSe}}{tr}\right)}^{1/2}$$

$$\;\text{III}.\;\text{SE}(gij)\;\mathrm{crosses}={\left(\frac{\text{MSe}}{r}\right)}^{1/2}$$

$$\;\text{IV}.\;\text{SE}(gj)\;\text{testers}={\left(\frac{\text{MSe}}{lr}\right)}^{1/2}$$

$$\;\text{V}.\;\text{SE}(gi-gj)\;\text{tester}={\left(\frac{2\text{MSe}}{lr}\right)}^{1/2}$$

$$\text{VI}.\;\text{SE}(sij-ski)\;\text{crosses}={\left(\frac{2\text{MSe}}{r}\right)}^{1/2}$$

#### Test of significance for gca and sca effects

$$\;\text{I}.\;Ti(\text{cal})\;\text{for gca of lines}=\left(\frac{gi-0}{\text{SE}(gi)}\right)$$

$$\text{II}.\;Tj(\text{cal})\mathrm{forgca}\text{of testers }=\left(\frac{gj-0}{\text{SE}(gj)}\right)$$

$$\text{III}.\;Tij(cal)\;\text{for sca}\;\text{of crosses}=\left(\frac{sij-0}{\text{SE}(sij)}\right)$$

The gca effects of lines and testers and sca effects of crosses were marked significant (*P < 0.05) and highly significant (**P < 0.01) when values of *Ti*, *Tj* and *Tij* were ≥‘*t*’ tabulated values at infinity (∞) error degree of freedom.

#### Biplot and path coefficient analysis

The genotype-by-trait (GT) biplot analysis was computed by following (Yan and Kang 2003):

$$\text{I}.\;\frac{\alpha_{ij}-\beta_{j}}{\sigma_{j}}=\sum_{n=1}^{2}\lambda_{n}\xi_{in}\eta_{\;jn}+\epsilon_{ij}=\sum_{n=1}^{2}\xi_{in}\eta_{\;jn}+\epsilon_{ij}$$

where *α_ij_* is the mean value of genotype *i* for trait *j*, *β_j_* the mean value of all genotypes for trait *j*, *σ_j_* the standard deviation of trait *j* among genotype means, *λ_n_* the singular value for principal component (PCn), *ξ_in_* the PCn score for genotype *i*, *η_jn_* the PCn score for trait *j* and *ε_ij_* is the residual associated with genotype *i* in trait *j*.

The path coefficient was performed following Dewey and Lu (1959). This technique involves partitioning of correlation coefficients to direct and indirect effects through alternate pathways of casual variables over resultant variables. *Bacillus thuringiensis*protein (Cry1Ac) was considered as resultant variable, while other studied traits were casual variables. The figures of path analysis were generated using PAST statistical packages (Hammer *et al*. 2001).

## Results

### Genetic effects and heritability estimates

There was significant variation among parents, parent vs. crosses, crosses and line × tester interaction for the biochemical traits (Table 1). The variance of gca was lower than the variance of sca for TSP, chlorophyll *a*, chlorophyll *b*, carotenoids, total chlorophyll, TPC and enzymatic antioxidants (SOD, POD and CAT) under control and drought conditions. The degree of dominance $(\sigma_{D}^{2}/\sigma_{A}^{2}{)}^{1/2}$ and $\sigma_{\mathrm{sca}}^{2}/\sigma_{\mathrm{gca}}^{2}$ ratio was greater than unity for all biochemical traits under both treatments. The amount of narrow sense heritabilities was low for all traits under both conditions, i.e. control and drought. Further, the narrow sense heritabilities under control were inconsistent with the narrow sense heritabilities under drought for all studied traits. The maximum amount of heritability was observed for carotenoids (20.28) and total chlorophyll (14.20) under control and drought conditions, respectively. The maternal genotypes (lines) were found superior for chlorophyll *a*, chlorophyll *b*, total chlorophyll and carotenoids, while the contribution of line × tester interaction was greater for TSP, TPC, SOD, POD and CAT under control treatment. However, contributions of paternal genotypes (tester) were lower for all biochemical traits. Under drought condition, a greater contribution of tester was recorded for carotenoids, and the contribution of lines was greater for chlorophyll *a*, chlorophyll *b*, total chlorophyll, TPC, SOD and POD, but for line × tester interaction, the contribution of TSP and CAT was higher (Table 2).

**Table 1.** Analysis of variance for biochemical traits under control and drought conditions in *Bt* cotton. SOV, source of variation; df, degrees of freedom; Tr, treatments; C, control; D, drought; MS, mean square; *F*, *F* ratio; TSP, total soluble protein; TPC, total phenolic contents; SOD, superoxide dismutase; POD, peroxidase; CAT, catalase.

SOV	df	Tr	TSP		Chlorophyll *a*		Chlorophyll *b*		Carotenoids		Total chlorophyll		TPC		SOD		POD		CAT	
			MS	*F*	MS	*F*	MS	*F*	MS	*F*	MS	*F*	MS	*F*	MS	*F*	MS	*F*	MS	*F*
Replications	2	C	2.14	0.31	0.38	2.00	0.16	3.13	0.04	3.71	0.20	0.39	0.05	1.65	464.71	1.08	794.22	11.66	23.35	2.81
		D	46.18	10.65	0.00	0.03	0.01	0.52	0.00	0.08	0.04	0.73	0.01	1.36	230.45	0.74	16.26	1.49	110.45	26.77
Genotypes	22	C	124.84	18.07	3.88	20.42	3.53	67.27	0.15	14.42	14.20	27.56	0.77	24.33	3 82 156.75	885.35	11 797.99	173.19	1272.24	153.38
		D	105.78	24.39	1.15	26.58	2.71	163.08	0.41	28.61	6.74	119.24	4.66	1317.31	198 754.82	634.76	22 817.12	2093.78	604.26	146.45
Parents	7	C	45.17	6.54	1.15	6.05	0.76	14.43	0.16	15.51	3.23	6.26	1.24	39.40	258 268.48	598.33	3115.49	45.73	534.01	64.38
		D	99.47	22.93	1.27	29.40	3.12	187.76	0.69	47.41	8.11	143.48	2.04	577.40	355 542.64	1135.50	32 115.24	2947.01	562.93	136.43
Parents vs. crosses	1	C	596.07	86.26	44.99	237.06	37.52	714.11	0.74	72.26	164.64	319.43	0.34	10.77	264 607.01	613.02	33 866.41	497.13	3389.14	408.60
		D	99.14	22.86	4.96	114.72	7.22	434.90	1.17	80.63	24.14	427.05	1.97	558.01	33 738.86	107.75	132 233.48	12 134.23	1603.05	388.53
Crosses	14	C	131.01	18.96	2.30	12.13	2.49	47.48	0.10	9.75	8.95	17.36	0.56	17.76	452 497.30	1048.31	14 562.94	213.77	1490.15	179.66
		D	109.41	25.22	0.82	18.87	2.18	131.33	0.22	15.49	4.81	85.13	6.16	1741.51	132 147.76	422.04	10 352.61	949.99	553.59	134.17
Lines	4	C	187.40	1.67	5.27	4.60	5.57	3.79	0.22	6.41	20.17	4.13	0.69	1.52	110 546.33	0.30	22 197.73	1.57	1109.78	0.76
		D	163.63	1.60	1.42	3.38	4.19	2.97	0.08	0.50	9.94	3.75	12.43	2.73	266 502.36	3.14	19 387.15	2.58	914.57	1.96
Testers	2	C	94.66	0.85	0.99	0.87	0.44	0.30	0.12	12.01	2.74	0.56	0.75	1.66	14 49 617.84	3.87	896.78	0.06	2350.83	1.60
		D	30.18	0.30	1.18	2.81	1.22	0.86	0.73	4.30	3.18	1.20	0.05	0.01	52 018.82	0.61	3581.16	0.48	177.27	0.38
Lines × testers	8	C	111.90	16.19	1.15	6.03	1.47	27.99	0.03	3.34	4.88	9.47	0.45	14.29	374 192.65	866.90	14 162.08	207.89	1465.16	176.64
		D	102.11	23.54	0.42	9.73	1.41	85.16	0.17	11.66	2.65	46.95	4.55	1287.40	85 002.68	271.47	7528.20	690.82	467.19	113.23
Error	44	C	6.91	–	0.19	–	0.05	–	0.01	–	0.52	–	0.03	–	431.65	–	68.12	–	8.30	–
		D	4.34	–	0.04	–	0.02	–	0.01	–	0.06	–	0.00	–	313.12	–	10.90	–	4.13	–

**Table 2.** Estimates of genetic components, heritabilities and per cent contribution of lines, testers and line × tester to the total variation for biochemical traits under control and drought conditions in *Bt* cotton. Tr, treatments; C, control; D, drought; $\sigma_{\mathrm{gca}}^{2}$, variance of gca; $\sigma_{\mathrm{sca}}^{2}$, variance of sca; $\sigma_{A}^{2}$, additive genetic variance; $\sigma_{D}^{2}$, dominant genetic variance; $(\sigma_{D}^{2}/\sigma_{A}^{2}{)}^{1/2}$, degree of dominance; *h*^2^(n.s), narrow sense heritability; df, degrees of freedom; TSP, total soluble protein; TPC, total phenolic contents; SOD, superoxide dismutase; POD, peroxidase; CAT, catalase.

Genetic components	Tr	TSP	Chlorophyll *a*	Chlorophyll *b*	Carotenoids	Total chlorophyll	TPC	SOD	POD	CAT
$\sigma_{\mathrm{gca}}^{2}$	C	0.68	0.04	0.04	0.00	0.14	0.00	2768.35	14.17	0.88
	D	0.26	0.01	0.03	0.00	0.08	0.06	1666.75	99.85	3.06
$\sigma_{\mathrm{sca}}^{2}$	C	34.99	0.32	0.47	0.01	1.46	0.14	124 587.00	4697.99	485.62
	D	32.59	0.13	0.47	0.05	0.87	1.52	28 229.86	2505.77	154.35
$\sigma_{\mathrm{gca}}^{2}/\sigma_{\mathrm{sca}}^{2}$	C	0.02	0.13	0.08	0.29	0.10	0.03	0.02	0.00	0.00
	D	0.01	0.11	0.06	0.04	0.09	0.04	0.06	0.04	0.02
$\sigma_{\mathrm{sca}}^{2}/\sigma_{\mathrm{gca}}^{2}$	C	51.81	7.78	13.06	3.45	10.13	36.03	45.00	331.51	549.77
	D	126.26	9.00	17.19	26.23	11.35	26.71	16.94	25.10	50.53
$\sigma_{A}^{2}$	C	1.35	0.08	0.07	0.01	0.29	0.01	5536.69	28.34	1.77
	D	0.52	0.03	0.05	0.00	0.15	0.11	3333.49	199.71	6.11
$\sigma_{D}^{2}$	C	34.99	0.32	0.47	0.01	1.46	0.14	124 587.00	4697.99	485.62
	D	32.59	0.13	0.47	0.05	0.87	1.52	28 229.86	2505.77	154.35
$(\sigma_{D}^{2}/\sigma_{A}^{2}{)}^{1/2}$	C	5.10	1.97	2.56	1.31	2.25	4.24	4.74	12.87	16.58
	D	7.95	2.12	2.93	3.62	2.38	3.65	2.91	3.54	5.03
*h*^2^(n.s)	C	3.12	13.87	12.12	20.28	12.72	4.33	4.24	0.59	0.36
	D	1.38	14.20	10.10	5.62	14.20	6.95	10.46	7.35	3.71
Contribution of lines	C	40.87	65.43	63.81	62.80	64.43	35.03	6.98	43.55	21.28
	D	42.73	49.82	54.95	10.73	59.04	57.65	57.62	53.51	47.20
Testers	C	10.32	6.15	2.50	17.61	4.38	19.02	45.77	0.88	22.54
	D	3.94	20.72	7.99	46.26	9.45	0.11	5.62	4.94	4.58
Lines × testers	C	48.81	28.42	33.69	19.59	31.19	45.96	47.25	55.57	56.19
	D	53.33	29.46	37.05	43.01	31.52	42.24	36.76	41.55	48.22

The estimates of gca effects of parents varied significantly for TSP, chlorophyll *a*, chlorophyll *b*, total chlorophyll, carotenoids, TPC and enzymatic antioxidants (SOD, POD and CAT) under control and drought conditions. Among lines, MNH-814 had maximum gca effects for TSP and SOD. The Egyptian cotton Giza-7 was found to have highest gca effects for chlorophyll *a*, chlorophyll *b*, total chlorophyll, carotenoids, TPC and CAT. The tester MNH-886 had the highest significant gca effects for carotenoids, TPC and POD. The line VH-303 showed maximum gca effects for TSP, SOD and CAT. The tester FH-142 was found better for chlorophyll contents under control condition, while under drought conditions, SA-1357 had maximum gca effects for carotenoids; MNH-814 had high gca effect for chlorophyll *a*, *b*, total chlorophyll contents and CAT; BZUG1 had high gca effect for SOD; BZUB had high gca effect for TSP and POD; and Giza-7 showed maximum gca effects for TPC. Among testers, MNH-886 was found better with maximum gca effects for TPC, SOD and CAT, and VH-303 showed maximum gca effects for TSP, total chlorophyll, chlorophyll *b* and POD, while FH-142 had highest gca effects for chlorophyll *a* and carotenoids (Table 3).

**Table 3.** General combining ability effects (gca) indicating the breeding value of lines and testers for biochemical traits under control and drought conditions in *Bt* cotton. *Significant at 5 % level of probability. **Significant at 1 % level of probability. Tr, treatments; C, control; D, drought; SE(*gi*), standard error (gca effects for lines); SE(*gi*–*gj*) line, standard error (between gca effects of two lines); SE(*gj*), standard error (gca effects for testers); SE(*gi*–*gj*) tester, standard error (between gca effects of two testers); TSP, total soluble protein; TPC, total phenolic contents; SOD, superoxide dismutase; POD, peroxidase; CAT, catalase.

	Tr	TSP	Chlorophyll *a*	Chlorophyll *b*	Carotenoids	Total chlorophyll	TPC	SOD	POD	CAT
Lines										
SA-1357	C	−3.56**	−0.26*	−0.75**	−1.01**	0.01	−0.05	44.94**	−31.49**	−15.22**
	D	−1.05	0.18*	−0.25**	−0.07	0.15**	−0.74**	7.49	−5.10**	−11.62**
MNH-814	C	5.58**	−0.96**	−0.72**	−1.68**	−0.18**	−0.28**	143.60**	−37.57**	0.22
	D	−0.79	0.52**	1.09**	1.62**	−0.11**	0.76**	−163.73**	−21.13**	9.49**
BZUG1	C	4.36**	0.29*	0.83**	1.13**	0.02	−0.24**	−15.40*	66.51**	1.34
	D	2.35**	−0.09	0.02	−0.07	0.01	−0.88**	185.49**	−5.17**	7.79**
BZUB	C	−3.26**	−0.19	−0.19*	−0.38	−0.09*	0.21**	−11.52*	−37.68**	−2.30
	D	5.46**	−0.56**	−0.77**	−1.33**	−0.05	−0.85**	153.82**	77.44**	4.41**
GIZA-7	C	−3.12**	1.11**	0.82**	1.93**	0.24**	0.35**	−161.62**	40.23**	15.96**
	D	−5.97**	−0.06	−0.09*	−0.14*	0.01	1.70**	−183.07**	−46.04**	−10.07**
SE(*gi*)	C	0.88	0.15	0.08	0.24	0.03	0.06	6.93	2.75	0.96
	D	0.69	0.07	0.04	0.08	0.04	0.02	5.90	1.10	0.68
SE(*gi*–*gj*) lines	C	1.24	0.21	0.11	0.34	0.05	0.08	9.79	3.89	1.36
	D	0.98	0.10	0.06	0.11	0.06	0.03	8.34	1.56	0.96
Testers										
MNH-886	C	−0.03	−0.25*	−0.17**	−0.42*	0.06*	0.25**	−202.47**	7.10**	−0.52
	D	0.47	−0.31**	−0.18**	−0.50**	−0.09**	0.06**	61.42**	−6.80**	2.14**
VH-303	C	2.53**	−0.01	−0.01	−0.02	−0.10**	−0.08*	357.94**	1.14	12.77**
	D	1.13*	0.08	0.33**	0.41**	−0.16**	−0.01	−55.98**	17.69**	−3.97**
FH-142	C	−2.50**	0.26*	0.17**	0.44*	0.04*	−0.17**	−155.46**	−8.24**	−12.25**
	D	−1.59**	0.23**	−0.15**	0.08	0.25**	−0.05**	−5.44	−10.88**	1.82**
SE(*gj*)	C	0.68	0.11	0.06	0.19	0.03	0.05	5.36	2.13	0.74
	D	0.54	0.05	0.03	0.06	0.03	0.02	4.57	0.85	0.52
SE(*gi*–*gj*) tester	C	0.96	0.16	0.08	0.26	0.04	0.06	7.59	3.01	1.05
	D	0.76	0.08	0.05	0.09	0.04	0.02	6.46	1.21	0.74

The intraspecific hybrid BZUB × VH-303 had highly significant positive sca effects for most of the studied traits, i.e. TSP, chlorophyll contents, carotenoids, SOD and POD. Similarly, the interspecific hybrid Giza-7 × FH-142 surpass all intraspecific hybrids with highest sca effects for TSP, SOD and POD under control condition, while the estimates of sca effects illustrated that intraspecific cotton hybrid BZUB × MNH-886 had significant sca effects for chlorophyll contents, carotenoids, TPC, POD and CAT. Similarly, cotton hybrids MNH-814 × FH-142 had significant sca effects for TSP, TPC, SOD, POD and CAT under drought condition (Table 4).

**Table 4.** Specific combining ability effects indicating genetic value of crosses due to interaction of their parents for biochemical traits in *Bt* cotton. *Significant at 5 % level of probability. **Significant at 1 % level of probability. SE(*ij*), standard error (sca effects for crosses); SE(*sij*–*ski*), standard error (between sca effects of two crosses); Tr, treatments; C, control; D, drought; TSP, total soluble protein; TPC, total phenolic contents; SOD, superoxide dismutase; POD, peroxidase; CAT, catalase.

Crosses	Tr	TSP	Chlorophyll *a*	Chlorophyll *b*	Carotenoids	Total chlorophyll	TPC	SOD	POD	CAT
SA-1357 × MNH-886	C	5.22**	−0.81**	−0.68**	−1.49**	−0.03	−0.18*	403.47**	61.78**	11.90**
	D	−1.89	0.23*	0.18*	0.41**	0.07	0.26**	1.58	−42.88**	−1.19
SA-1357 × VH-303	C	−3.75*	0.73**	0.79**	1.53**	−0.04	0.41**	−197.94**	−26.70**	−16.10**
	D	3.88**	−0.22*	−0.46**	−0.68**	0.04	0.82**	33.64**	51.76**	3.62*
SA-1357 × FH-142	C	−1.47	0.08	−0.11	−0.03	0.07	−0.22*	−205.54**	−35.09**	4.19*
	D	−1.99*	−0.01	0.27**	0.26*	−0.12*	−1.08**	−35.22**	−8.88**	−2.43*
MNH-814 × MNH-886	C	5.68**	0.71**	0.60**	1.31**	−0.03	0.04	−142.19**	−10.15*	2.76*
	D	−7.40**	−0.01	−0.14*	−0.15	0.01	0.21**	−129.53**	−45.78**	−17.74**
MNH-814 × VH-303	C	−3.77*	−0.29	−0.28*	−0.58	0.05	−0.02	405.40**	3.09	−2.64
	D	−2.02*	0.18	0.79**	0.98**	−0.08	−1.08**	−97.47**	−26.31**	−4.91**
MNH-814 × FH-142	C	−1.91	−0.42*	−0.32*	−0.73*	−0.03	−0.01	−263.20**	7.06	−0.12
	D	9.42**	−0.18	−0.65**	−0.83**	0.07	0.87**	227.00**	72.09**	22.65**
BZUG1 × MNH-886	C	0.93	0.1	0.27*	0.38	0.05	0.56**	−99.19**	41.82**	20.14**
	D	6.87**	−0.05	0.39**	0.34*	−0.28**	0.75**	−67.42**	21.29**	11.59**
BZUG1 × VH-303	C	2.98*	−0.55*	−1.08**	−1.63**	0.03	−0.15	−38.60**	43.46**	−14.52**
	D	−1.60	−0.28*	−0.32**	−0.60**	−0.01	−0.22**	171.98**	20.34**	1.40
BZUG1 × FH-142	C	−3.91*	0.45*	0.81**	1.26**	−0.05	−0.41**	137.80**	−85.29**	−5.62**
	D	−5.27**	0.33**	−0.07	0.26*	0.29**	−0.53**	−104.56**	−41.63**	−12.99**
BZUB × MNH-886	C	−1.95	−0.11	−0.28*	−0.39	−0.06	−0.36**	−185.45**	−9.79*	3.38*
	D	0.29	0.33**	0.50**	0.84**	0.20**	0.33**	−51.09**	25.22**	5.31**
BZUB × VH-303	C	2.81*	0.47*	0.48**	0.95*	0.15*	−0.14	265.52**	9.85*	−3.40*
	D	2.40*	0.15	−0.68**	−0.53**	0.18*	−1.17**	−19.36*	−1.87	0.51
BZUB × FH-142	C	−0.86	−0.36	−0.20	−0.57	−0.09	0.51**	−80.08**	−0.06	0.02
	D	−2.68*	−0.49**	0.18*	−0.31*	−0.38**	0.84**	70.44**	−23.35**	−5.81**
GIZA-7 × MNH-886	C	−9.89*	0.11	0.09	0.19	0.06	−0.06	23.36*	−83.66**	−38.18**
	D	2.12*	−0.50**	−0.93**	−1.43**	0.03	−1.56**	246.47**	42.15**	2.02
GIZA-7 × VH-303	C	1.73	−0.36	0.09	−0.27	−0.16**	−0.08	−434.38**	−29.71**	36.65**
	D	−2.65*	0.16**	0.66**	0.82**	−0.13*	1.66**	−88.80**	−43.92**	−0.61
GIZA-7 × FH-142	C	8.15*	0.25	−0.17	0.08	0.09*	0.14	411.02**	113.38**	1.53
	D	0.53	0.34**	0.27**	0.61**	0.14*	−0.10**	−157.67**	1.77	−1.41
SE(*ij*)	C	1.52	0.25	0.13	0.41	0.06	0.1	12.00	4.77	1.66
	D	1.20	0.12	0.07	0.14	0.07	0.03	10.22	1.91	1.17
SE(*sij*–*ski*)	C	2.15	0.36	0.19	0.59	0.08	0.14	16.96	6.74	2.35
	D	1.70	0.17	0.11	0.19	0.10	0.05	14.45	2.70	1.66

### Biplot analysis

**Figure 1.**
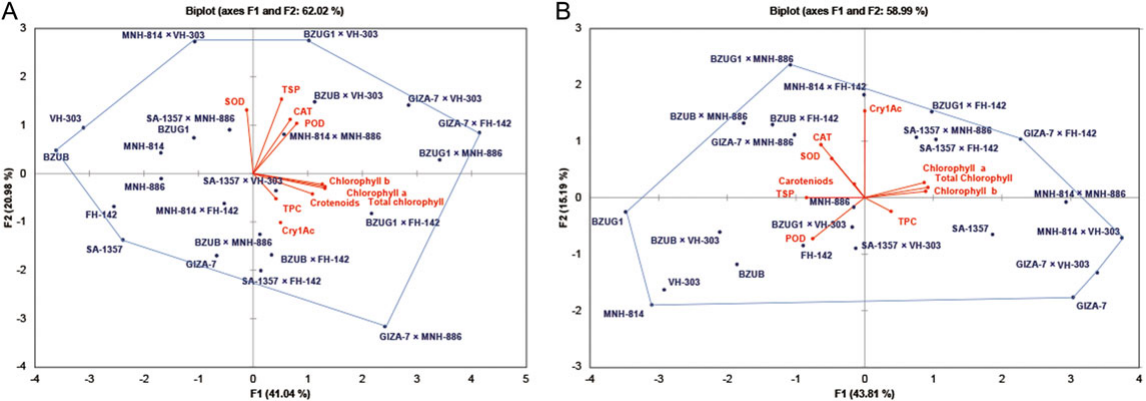
Biplot analysis for biochemical traits in *Bt* cotton. (A) Control and (B) drought conditions. TSP, total soluble protein; TPC, total phenolic contents; SOD, superoxide dismutase; POD, peroxidase; CAT, catalase.

The angle between trait vectors of *Bt* toxin (Cry1Ac) and other biochemical traits except SOD, POD, CAT and TSP was <90 ° under control (Fig. 1A). However, the trait vector of *Bt* toxin Cry1Ac had a >90 ° angle with TSP, TPC and POD under drought condition (Fig. 1B).

The identification and evaluation of elite cotton genotypes for different biochemical markers under both stressed and non-stressed conditions was done by GT biplot analysis. Genotype by trait biplot analysis depicted that three genotypes VH-303, BZUB and SA-1357; interspecific hybrids (Giza-7 × MNH-886 and Giza-7 × FH-142) and two intraspecific hybrids BZUG1 × VH-303 and MNH-814 × VH-303 were at the vertex of the polygon under control condition (Fig. 1A). Among these hybrids, BZUG1 × VH-303 and MNH-814 × VH-303 were found near the trait vectors of TSP, SOD, POD and CAT, and a hybrid SA-1357 × VH-303 was near to the origin of biplot. However, two interspecific hybrids Giza-7 × MNH-886 and Giza-7 × FH-142 were found farthest from the origin but at or near the trait vectors of total chlorophyll contents, TPC, carotenoids and Cry1Ac under control condition (Fig. 1A). Similarly, the GT biplot analysis revealed that intraspecific hybrids (BZUG1 × MNH-886, BZUG1 × FH-142 and MNH-814 × VH-303) and two interspecific hybrids Giza-7 × FH-142, and Giza-7 × VH-303 along with three genotypes, i.e. Giza-7, MNH-814 and BZUG1, were at the vertex of the polygon under drought condition. Among these cotton hybrids, BZUG1 × MNH-886 and BZUG1 × FH-142 were observed near the trait vectors of Cry1Ac, SOD and CAT. Parental genotypes, viz. MNH-814 and BZUG1, were found close to the trait vectors of TSP and POD (Fig. 1B).

### Path coefficient analysis

**Figure 2.**
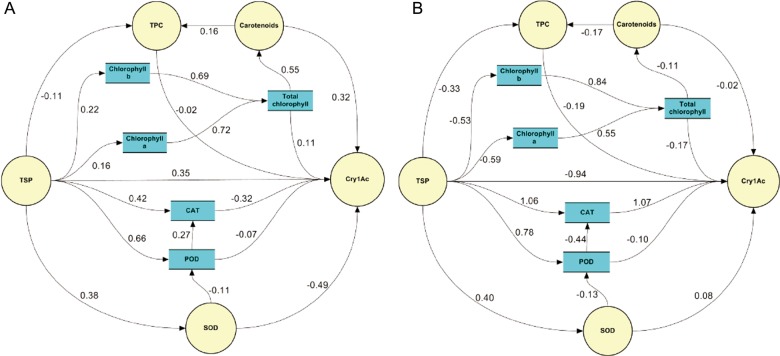
Path coefficient analysis for biochemical traits in *Bt* cotton. (A) Control and (B) drought conditions. TSP, total soluble protein; TPC, total phenolic contents; SOD, superoxide dismutase; POD, peroxidase; CAT, catalase.

The total chlorophyll contents, carotenoids and TSP had positive direct effects on Cry1Ac toxin under control conditions, while the TPC, SOD, POD and CAT had negative direct effects on Cry1Ac toxin. Results also depicted that the TSP had a positive direct effect on chlorophyll *a*, chlorophyll *b*, SOD, POD and CAT but had a negative direct effect on TPC. In addition, TSP had an indirect negative effect on POD via SOD. The positive indirect effect of chlorophyll *a* and *b* on carotenoids and Cry1Ac toxin was observed under control condition (Fig. 2A). The TSP, total chlorophyll contents and chlorophyll *a* and *b* had negative direct and indirect effects on Cry1Ac toxin under drought condition. However, TSP had direct positive effect on SOD, POD and CAT and negative effect on TPC and chlorophyll *a* and *b*, respectively (Fig. 2B).

## Discussion

*Bacillus thuringiensis* cotton is the product of modern agricultural research that is continuously replacing the cultivation of non-*Bt* cotton cultivars. Different *Bt* cotton genotypes have different abilities to tolerate drought by varying the expression of various biochemical traits according to their genetic potential. It has also been reported that drought is known to affect the efficacy of transgenes in genetically modified crops (Bruns and Abel 2003). Therefore, exploring the mode of inheritance for the various biochemical traits analysed in this study will enable plant breeders to develop *Bt* cotton genotypes having a desired amount of *Bt* toxin and antioxidant activity under water stressed and non-stressed conditions.

Non-additive gene action was involved in the expression of all biochemical traits including TSP, chlorophyll *a*, chlorophyll *b*, carotenoids, total chlorophyll, TPC and enzymatic antioxidants (SOD, POD and CAT) under control and drought conditions. These findings are confirmed by the reports of Mehndiratta and Phul (1983), Greish *et al.* (2005) and Immanuel *et al.* (2006) in pearl millet, tomato and maize, respectively. Our findings also suggested that heterosis breeding would be more fruitful for the development of drought-tolerant *Bt* cotton hybrids. However, Song *et al.* (2014) reported the preponderance of additive genetic effects for SOD and POD activity in cotton. The difference in gene action could be attributed to environmental factors and different genetic make-up of breeding material.

Heritability is a good index for transmission of traits from parents to offspring, and the scope of trait improvement through selection breeding depends upon the magnitude of heritability (Neelima and Chenga 2008). In our findings, low amount of narrow sense heritability coupled with higher degree of dominance $(\sigma_{D}^{2}/\sigma_{A}^{2}{)}^{1/2}$ and ratio $\sigma_{\mathrm{sca}}^{2}/\sigma_{\mathrm{gca}}^{2}$ for all biochemical traits under both conditions further confirmed the prevalence of non-additive genetic effects. These findings demonstrated that selection based on all these biochemical traits in early segregating generations would be less efficient (Saleem *et al.* 2009; Rad *et al.* 2012). The low amount of heritabilities for different biochemical traits also suggested that direct selection based on these traits will not yield encouraging results as they are influenced not only by genotype but also by environment and genotype × environment interaction (Rosielle and Hamblin 1981; Ribaut *et al*. 1997).

Estimates of combining ability demonstrated the breeding value of parental lines towards the development of a desired genotype. General combining ability is due to additive-type gene action, whereas sca is administrated by the non-additive nature of genes (Wu *et al.* 2006; Jenkins *et al.* 2007). In the present study, parental lines, viz. MNH-814, GIZA-7, MNH-886 and FH-142, with highly significant gca effects for biochemical traits, i.e. TSP, chlorophyll *a*, chlorophyll *b*, total chlorophyll, carotenoids, TPC and enzymatic antioxidants (SOD, POD and CAT), suggested that these genotypes had more tendency to pass these biochemical traits to their progenies under control conditions (Rad *et al.* 2012), whereas parental genotypes, i.e. MNH-814, BZUG1, BZUB, MNH-886 and VH-303, with more gca effects were found superior combiners for chlorophyll *a*, *b*, total chlorophyll contents, TSP and enzymatic antioxidants under drought. Our findings also suggested that parents with high gca effects for specific traits might have additive gene action and these parents could be used for the development of genotypes having greater amounts of these biochemical traits through hybridization followed by selection breeding (Saleem *et al*. 2009). Specific estimates of combining abilities showed that intraspecific hybrid (BZUB × VH-303) and interspecific hybrid (GIZA-7 × FH-142) were found best combiners for TSP, chlorophyll contents, carotenoids, SOD, POD and CAT under control condition, whereas BZUB × MNH-886 and MNH-886 × FH-142 were best specific combiners for chlorophyll contents, carotenoids, TPC, TSP, SOD, POD and CAT under drought conditions. The results suggested that these hybrids represent an ample scope for hybrid development of *Bt* cotton with more tolerance to drought (Ashokkumar *et al.* 2010). In our study, the hybrids with negative value of sca for different biochemical traits under both conditions, i.e. normal and drought, indicated the existence of different genes with minor effects in each line or prevalence of epistasis (Abd *et al.* 2009).

The correlation coefficient among various traits can be estimated using biplot analysis. In this biometrical technique, a trait vector is drawn from the origin to each marker of the traits, and cosine of the angle among trait vectors determine the nature of association among them (Yan and Rajcan 2002), whereas for traits having a multidirectional relationship with each other, path analysis could be a useful biometrical tool in predicting correlation responses to directional selection and in identifying traits that may not be important from a breeding point of view but that can serve as precursors for the important ones (Rasheed *et al*. 2009). The hybrids at the vertex and near any trait vector could be attributed best or poor for that particular trait (Yan *et al.* 2007). In biplot analysis, those hybrids that were found to be best for different biochemical traits under control and drought conditions suggested that better performance of these hybrids when compared with their parents might be due to accumulation of favourable alleles and increase in heterozygosity at different loci, because heterozygosity is always superior to homozygosity (Springer and Stupar 2007). In our study, the positive association coupled with positive direct effects among various biochemical traits suggested that the prevalence of pleiotropic gene effects or linkage and selection based on these traits will lead to simultaneous improvement in these biochemical traits (Iqbal *et al*. 2003). However, negative association and negative direct effect among different traits can be broken by random mating leading to rigorous selection breeding (Miller and Rawlings 1967).

The biochemical basis of negative associations between Cry1Ac toxin with TSP under control and drought conditions and negative association coupled with negative direct effect of enzymatic antioxidant (SOD, POD and CAT) on Cry1Ac protein only under control conditions suggested that all available nitrogen might be routed for enzymes and protein synthesis associated with growth and survival therefore cannot be allocated to toxin (Cry1Ac) synthesis (Coviella *et al*. 2002). However, the positive association and positive direct effects of SOD and CAT over Cry1Ac toxin under drought conditions signify the capability of eliminating the free radicals of reactive oxygen by the *Bt* cotton. In our study, negative association and negative direct effect of TPC with TSP and *Bt* toxin could be supported by the ‘Protein Competition Model of phenolic allocation’. According to this model, the metabolic pathways of plants allocated to either phenolics or soluble proteins compete for a common precursor phenylalanine, which acts as limiting factor (Jones and Hartley 1999). In this study, our finding regarding negative association of chlorophyll contents with soluble protein under control and drought conditions might be attributed to the concept that plants are able to shift allocation between carbon-based and nitrogen-based defensive compounds depending upon the availability of carbon and nitrogen nutrients (Coviella *et al.* 2002). Similarly, positive direct effects and positive association of chlorophyll *a*, chlorophyll *b*, total chlorophyll and carotenoids with Cry1Ac toxin under control illustrated that amount of these biochemical traits corresponds to the amount of Cry1Ac protein (Hosagoudar *et al.* 2008; Poongothai *et al.* 2010).

## Conclusions

The mode of inheritance for various biochemical traits suggest that there is room for the improvement of these traits under water stressed and non-stressed conditions. The biplot and path coefficient analyses proved to be effective biometrical tools in revealing that biochemical traits behaved differentially with Cry1Ac toxin under control and drought conditions. The differential behaviour of these biochemicals with Cry1Ac suggested that these traits can serve as biochemical markers while breeding *Bt* cotton. Further, critical understanding about inheritance and the association between different biochemical traits are likely to pave the way for breeding cotton genotypes having a desired level of Cry1Ac toxin with ample tendency to withstand drought conditions.

## Sources of Funding

This study was supported by the research grant for Department of Plant Breeding and Genetics, Bahauddin Zakariya University, Multan.

## Contributions by the Authors

M.A.A., W.M. and A.Y. conceived and design the study. M.A.A. and W.M. conducted the experiments. All authors contributed in data analysis and manuscript preparation.

## Conflict of Interest Statement

None declared.
